# Supplementary material for: The interactome of intact mitochondria by cross-linking mass spectrometry provides evidence for coexisting respiratory supercomplexes
Source: Mol Cell Proteomics. 2017 Dec 8;17(2):216–32. doi: 10.1074/mcp.RA117.000470 (PMC5795388; doi:10.1074/mcp.RA117.000470)
Supplement: Supplemental Data [file supp_RA117.000470_133922_0_supp_23978_fzffwf.zip › spectra_annotation/mito_spectra_annotation/225-1-11-1-6-1.pdf]

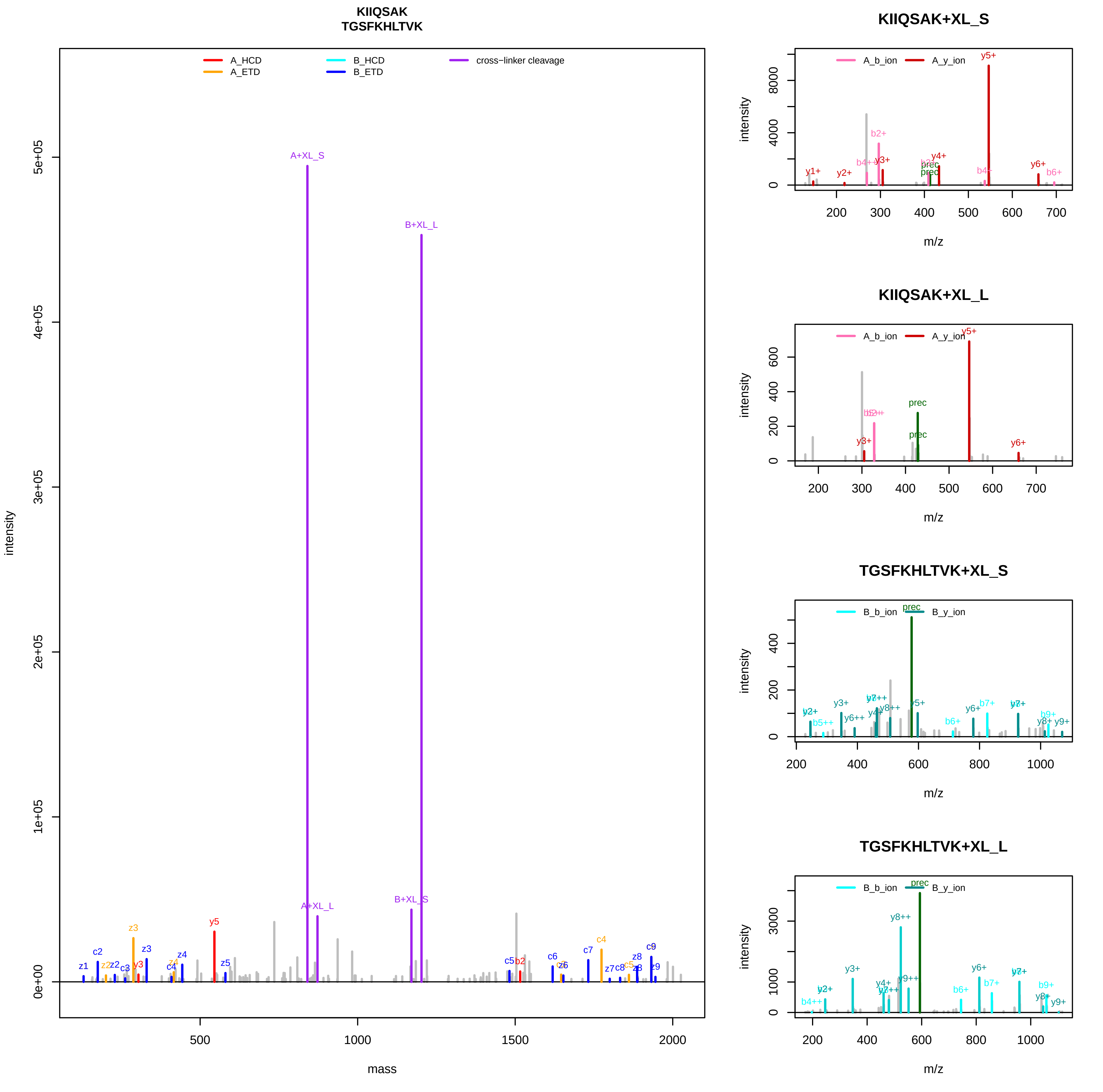
KIIQSAK
TGSFKHLTVK
A_HCD
A_ETD
B_HCD
B_ETD
cross−linker cleavage
A+XL_S
B+XL_L
A+XL_L
B+XL_S
y5
z3
c2
z1
z2
c3
y3
z4
c4
z5
c5
b2
c6
z6
c7
c4
z7
c8
c5
z8
c9
z9
intensity
mass
0e+00
1e+05
2e+05
3e+05
4e+05
5e+05
500
1000
1500
2000
KIIQSAK+XL_S
A_b_ion
A_y_ion
y5+
b2+
y3+
y4+
y6+
b6+
y1+
y2+
prec
KIIQSAK+XL_L
prec
y3+
y6+
m/z
TGSFKHLTVK+XL_S
B_b_ion
B_y_ion
prec
y3+
y5+
y6+
b7+
b6+
b9+
y9+
TGSFKHLTVK+XL_L
y8++
y3+
y9++
y6+
b6+
b7+
b4++
y2+
b9+
y9+
